# 97333 The NYU Langone Annual Health Disparities Symposium

**DOI:** 10.1017/cts.2021.603

**Published:** 2021-03-30

**Authors:** Smiti Nadkarni, Janet Pan, Nadia Islam, Simona Kwon, Antoinette Schoenthaler, Joseph Ravenell

**Affiliations:** NYU-H+H Clinical & Translational Science Institute

## Abstract

ABSTRACT IMPACT: This poster will demonstrate how input from a CTSI Community Advisory Board was used to develop a large, annual dissemination event focused on health disparities, health equity, and community engagement. OBJECTIVES/GOALS: The NYU Langone Annual Health Disparities Symposium began in response to the NYU-H+H CTSI’s Community Advisory Board, which expressed a desire to 1) learn about health disparities research at NYU, H+H, and beyond; 2) build connections and interdisciplinary collaborations; 3) support bidirectional dissemination between community and researchers. METHODS/STUDY POPULATION: The annual symposium, a collaboration between NYU Langone’s CTSI, Department of Population Health, Office of Diversity Affairs, and the NYU-CUNY Prevention Research Center, features a keynote, a series of rapid-fire talks, panels on current controversies in population health and the work of the Community Engagement Cores of NYC-based CTSAs, and poster sessions. Each year the event is focused around a specific theme, with the 2020 theme being ‘Research Into Action’. Audience members include faculty, staff, students, health care providers, community health workers, and representatives from community-based organizations, health care facilities, and the NYC Department of Health and Mental Hygiene. For the very first time, the event was held virtually days and CME/CNE credits were provided free of cost. RESULTS/ANTICIPATED RESULTS: The conference explored how institutions have turned research into action, and speakers addressed the ways in which COVID-19 has highlighted structural inequities that have existed across time. 585 attendees participated in the event, with 63 claiming an average of 7.8 hours of continuing education credits. 46 individuals completed the post-event evaluation, with 95% agreeing/strongly agreeing that the symposium increased their awareness of health disparities research taking place at NYU, H+H, and beyond, 91% agreeing/strongly agreeing that they are likely to apply the information learned to their own work, and 91% agreeing/strongly agreeing that the symposium increased their interest in health disparities research. 86% were very/extremely satisfied with the quality of the meeting overall. DISCUSSION/SIGNIFICANCE OF FINDINGS: The 2020 event had the greatest proportion of health care provider attendees (24%), likely due to the opportunity to earn CME/CNE credits. Attendance also grew over the years, from 150 in 2015 to 585 in 2020. This increase is likely due to increased awareness of the event, as well as well as virtual the format, which made it more convenient for attendees.

